# Trends in Topical Prescriptional Therapy for Old Patients With Dry Eye Disease in Six Major Areas of China: 2013–2019

**DOI:** 10.3389/fphar.2021.690640

**Published:** 2021-08-10

**Authors:** Zhenwei Yu, Xiaoyan Wu, Jianping Zhu, Jiayi Jin, Yuhua Zhao, Lingyan Yu

**Affiliations:** ^1^ Department of Pharmacy, Sir Run Run Shaw Hospital, Zhejiang University School of Medicine, Hangzhou, China; ^2^ Department of Pediatrics, Shaoxing Shangyu People’s Hospital of Shaoxing, Shaoxing, China; ^3^ Biomedical Research Center, Sir Run Run Shaw Hospital, Zhejiang University School of Medicine, Hangzhou, China; ^4^ Department of Pharmacy, Affiliated Xiaoshan Hospital, Hangzhou Normal University, Hangzhou, China; ^5^ Department of Pharmacy, Second Affiliated Hospital, Zhejiang University School of Medicine, Hangzhou, China

**Keywords:** dry eye, eye drop, ocular lubricant, artificial tear, sodium hyaluronate, prescription

## Abstract

The prevalence of dry eye disease (DED) in old patients are high, corresponding to a substantial economic burden. In this cross-sectional study, we analyzed the trends in the topical prescriptional treatment of old patients with DED in six major areas of China. Information on topical drug prescriptions for DED patients aged above 60 years was extracted from the Hospital Prescription Analysis Cooperative Program of China database. Trends in yearly prescriptions and cost were analyzed. The data were further stratified by patient age and sex, drug class, and specific drug. A total of 130,734 prescriptions from 52 hospitals located in six major areas of China were analyzed. The number of prescripptions per year for patients with DED increased from 13,308 in 2013 to 22,074 in 2019, with a corresponding increase in cost of all topical drugs from 1,490,014 Chinese Yuan (CNY) to 2,618,206 CNY. Drugs for the treatment of DED accounted for the largest proportion of the total cost in each year. Ocular lubricants were the main pharmacotherapy agent. Sodium hyaluronate use increased over time, and the drug was used by 65.9% of patients by the end of the study. Pranoprofen was the second most frequently used drug. The most frequently used drugs for co-incident disease were antimicrobials. Treatment patterns for DED haven’t changed, and the most frequently used drug combination was sodium hyaluronate and pranoprofen. In summary, prescription for old patients with DED and the cost of treatment are increasing. Ocular lubricants are the main treatment option, while sodium hyaluronate is the most frequently used drug. The observed trends can lead to more efficient allocation of health care resources in China.

## Introduction

Dry eye disease (DED) is a common multifactorial ocular surface disorder characterized by eye discomfort, disabling pain, and fluctuating vision, which can affect vision-related quality of life and reduce working time (Clayton, 2018). The precise etiology of DED is unclear, but it may be caused or exacerbated by multiple factors including medications, contact lenses, ocular surgery, computer use, and low-humidity environments (Clayton, 2018). The prevalence of DED varies by country, but all show an increasing trend (Courtin et al., 2016; Dana et al., 2019b; Siffel et al., 2020; Stapleton et al., 2017). It is reported that the prevalence of DED by symptoms and signs were 13.55% in Chinese people, corresponding to a total of 170.09 million affected individuals (Song et al., 2018). Advanced age is positively associated with an increased prevalence among people (Farrand et al., 2017; Song et al., 2018). For people aged over 60 years, the prevalence raised to 34.4% (Liu et al., 2014). Thus, DED affects the life quality of old patients substantially (McDonald et al., 2016). More public health attention and action are needed to improve the management of DED.

DED can be treated but not cured; the goal of treatment is to increase the patient’s quality of life by reducing symptoms (Marshall and Roach, 2016). Management strategies should consider the cause and severity of the disease and address the various disease components (Clayton, 2018). There are many classes of drug on the market for DED treatment including ocular lubricants, anti-inflammatory drops, essential fatty acids, and so on. Topical formulations offer several advantages such as simple, convenience and painless use (Agarwal et al., 2021). However, many treatments are poorly supported by evidence-based practices (Jones et al., 2017), and the efficacy of some (e.g., cyclosporine) is debated (Seitzman and Lietman, 2018). Pharmacotherapeutic approaches also vary by country because of differences in the understanding of DED etiology (Waduthantri et al., 2012; Watanabe, 2018). Ocular lubricant formulations such as sodium hyaluronate drops are favored by those who attribute DED to insufficient tear production (Watanabe, 2018). To date, there have been few reports on the usage of topical treatments for old patients with DED; however, greater awareness of the trends can improve health care resource utilization (McDonald et al., 2016). To address this issue, we carried out a cross-sectional study in six major areas to assess the trends in topical prescriptional pharmacotherapy for old patients with DED in China from 2013 to 2019.

## Methods

### Study Design

This prescription-based cross-sectional study was approved by the Ethics Committee of Sir Run Run Shaw Hospital, Zhejiang University School of Medicine (Reference number, 20191011–18). The requirement for informed consent was waived as part of the approval because of the retrospective nature of the study.

### Data Source and Study Population

Prescription data were extracted from the database of the Hospital Prescription Analysis Cooperative Program, which has been widely used in Chinese pharmaco-epidemiology studies (Yu et al., 2019; Yu et al., 2020a; Yu et al., 2020b; Yu et al., 2020c; Yu et al., 2020d; Yu et al., 2020e). Participating hospitals provided data on prescriptions to the program for each sampling day. There were forty randomized sampling days per year, with 10 days in each quarter. Prescription data included the date, patients’ code, sex, age, and diagnosis, as well as the generic name and price of the prescribed drug.

Prescription data from fifty-two hospitals in Beijing, Hangzhou, Chengdu, Guangzhou, Shanghai, and Tianjin were selected. These hospitals participated continuously in the program from 2013 to 2019 and were located in the north, west, south, and east of China, thus covering a wide geographic area and yielding data representative of the whole country. Prescriptions meeting the following criteria were included: 1) prescriptions for patients with a diagnosis of DED, with no restrictions regarding diagnostic criteria and disease severity; 2) prescriptions for patients aged 60 years old and above; 3) prescriptions written by an ophthalmologist between 2013 and 2019; and 4) prescriptions for at least one topical drug. Prescriptions with incomplete information were excluded from the analysis.

### Assessment of Drug Use

Only topical ocular medications were assessed in this study. Prescriptions were divided into drugs for the treatment of DED and those for co-incident diseases. The following types of drug were used for DED treatment: 1) ocular lubricants; 2) nonsteroidal anti-inflammatory drugs (NSAIDs); 3) corticosteroid; 4) vitamin A preparation; and 5) immunosuppressant (Jones et al., 2017). Drugs for the treatment of co-incident disease included the following: 1) anti-microbial agents; 2) growth factor preparations; 3) anti-allergy drugs; 4) glaucoma drugs; 5) cataract drugs; 6) complementary drugs; and 7) other.

Drug usage was assessed by prescription numbers, irrespective of whether it was new or a refill, and cost. Cost was calculated by adding the price of all analyzed drugs in Chinese Yuan (CNY). Trends in yearly prescriptions and cost were analyzed and further stratified by sex, age, drug class, and specific drug. The treatment pattern was classified as monotherapy or combined therapy with drugs for DED treatment.

### Statistical Analysis

Data were processed using Access software (Microsoft, Redmond, WA, United States). The rank-sum test was used to evaluate the statistical significance of overall trends in prescriptions and cost; the chi-squared test was used to compare prescriptions in males vs. females in each year; and the Cochran-Armitage trend test was used to assess trends in prescribed drugs and drug classes. Trends in percentages were assessed by log-linear analysis. All statistical analyses were performed using R v.3.3.0 software (http://www.R-project. org). A *p* value < 0.05 was considered statistically significant.

## Results

### Inclusion of Prescriptions and Overall Trends in Prescriptions and Cost

**FIGURE 1:**
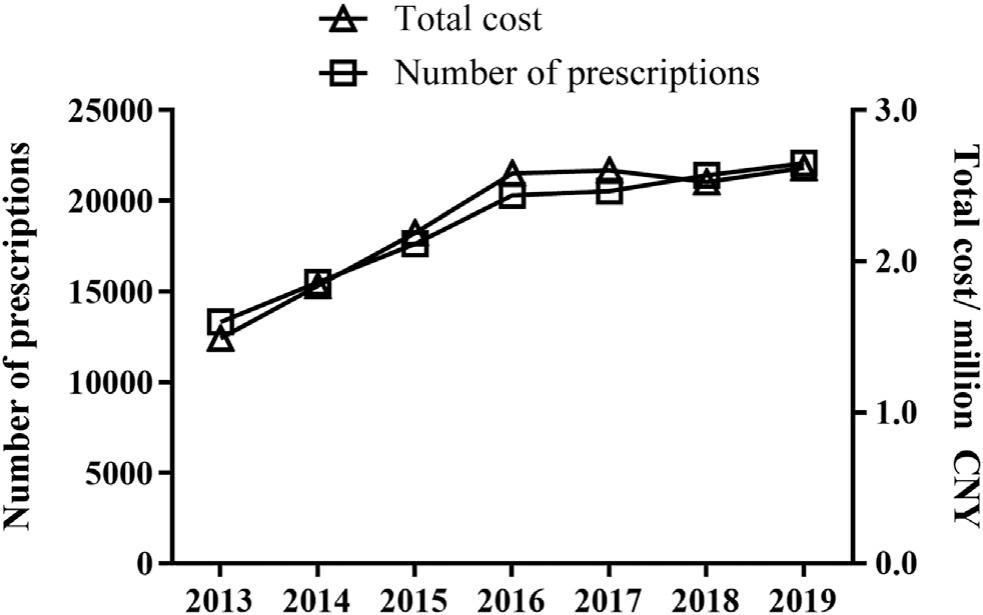
Trends in prescription numbers and cost of topical drugs for DED treatment. Cost was calculated in Chinese Yuan.

A total of 130,734 prescriptions from 52 hospitals were included in the analysis. All included hospitals were state owned. Of these, 48 were tertiary hospitals and eight were secondary hospitals. Yearly prescription for old patients with DED increased markedly from 13,308 in 2013 to 22,074 in 2019 (*p* < 0.05) (Figure 1). The corresponding cost also increased from CNY 1,490,014 to CNY 2,618,206 (*p* < 0.05).

The demographic information of old DED patients for the included prescriptions is shown in Table 1. Nearly half of the patients were between sixty and 70 years of age. Moreover, the proportion of patients at this age increased over time (*p* < 0.05). The prescriptions of female were more than the prescriptions of male in each year (Chi-square test, all *p* < 0.05).

**TABLE 1:** Demographic information of old DED patients for the included prescriptions.

	Number of patients (%)							P_1_	P_2_
	2013	2014	2015	2016	2017	2018	2019		
Age (years)									
61–70	6,381 (47.9)	7,612 (49.1)	8,776 (49.8)	10,333 (50.9)	10,458 (51.0)	11,125 (52.0)	11,671 (52.9)	0.003	<0.001
71–80	5,054 (38.0)	5,477 (35.3)	6,025 (34.2)	6,415 (31.6)	6,405 (31.2)	6,385 (29.8)	6,513 (29.5)	0.036	<0.001
80 up	1873 (14.1)	2,422 (15.6)	2,835 (16.1)	3,542 (17.5)	3,658 (17.8)	3,884 (18.2)	3,890 (17.6)	0.003	0.006
Sex									
Male	4,744 (35.6)	5,414 (34.9)	6,117 (34.7)	7,077 (34.9)	7,052 (34.4)	7,252 (33.9)	7,451 (33.8)	0.007	0.084
Female	8,564 (64.4)	10,097 (65.1)	11,519 (65.3)	13,213 (65.1)	13,469 (65.6)	14,142 (66.1)	14,623 (66.2)	0.003	0.084
Total	13,308	15,511	17,636	20,290	20,521	21,394	22,074	0.003	-

P_1_, *p*-value for trend in number of prescriptions, assessed by Mann–Kendall trend test; P_2_, *p*-value for trend in proportion of prescriptions, assessed by log-linear analysis.

### Trends in Drugs for DED Treatment

**FIGURE 2:**
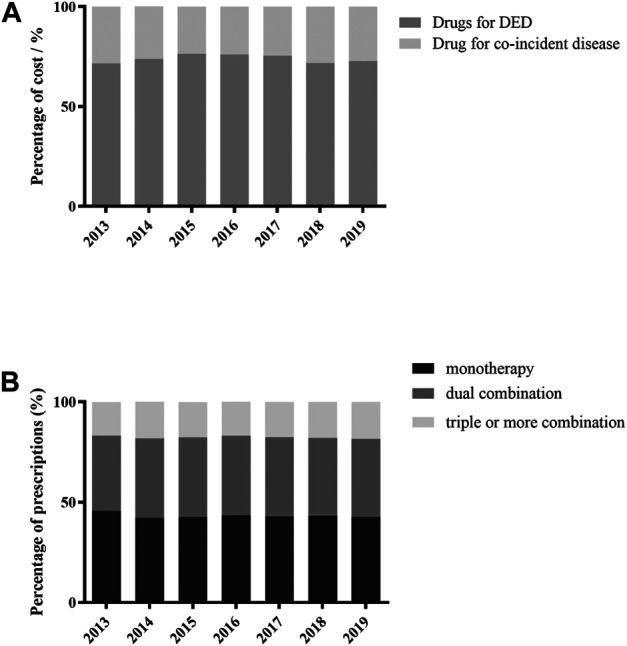
Trends in drug classes for DED treatment. **(A)** Trends in the costs of drugs for the treatment of DED and co-incident disease. **(B)** Trends in patterns of DED topical treatment.

Drugs for DED treatment accounted for more than 70 percent of the total prescription cost of topical drugs prescribed to old patients with DED (Figure 2A). Yearly prescription and cost of each drug are shown in Tables 2 and 3. The main drugs were ocular lubricant formulations containing sodium hyaluronate, polyvinyl alcohol (PVA), hydroxypropyl methyl cellulose (HPMC), carboxymethyl cellulose (CMC), and polyethylene glycol (PEG). Sodium hyaluronate was the most frequently prescribed ocular lubricant formulation and was used by more than half of the patients. Prescriptions for sodium hyaluronate and the corresponding cost both showed increasing trends over time (both *p* < 0.05). PVA drops were the second most frequently prescribed ocular lubricant formulation at the end of study. Meanwhile, the other three lubricants (HPMC and CMC and PEG) were used by progressively lesser percentages of patients (all *p* < 0.05).

**TABLE 2:** Prescription for topical drugs for the treatment of dry eye disease.

Drug class	Drug	2013	2014	2015	2016	2017	2018	2019	P_1_	P_2_
Lubricant	Sodium hyaluronate	7,013 (52.7)	7,831 (50.5)	9,322 (52.9)	11,297 (55.7)	11,786 (57.4)	13,759 (64.3)	14,544 (65.9)	0.003	0.002
	PVA	1,174 (8.8)	1770 (11.4)	2,340 (13.3)	2,837 (14.0)	2,705 (13.2)	2,667 (12.5)	2,667 (12.1)	0.172	0.190
	HPMC	1,628 (12.2)	1894 (12.2)	1868 (10.6)	1877 (9.3)	1,647 (8.0)	849 (4.0)	775 (3.5)	0.133	0.002
	CMC	1,045 (7.9)	2082 (13.4)	2,165 (12.3)	1,657 (8.2)	1,486 (7.2)	999 (4.7)	732 (3.3)	0.133	0.027
	PEG	972 (7.3)	1,033 (6.7)	1,169 (6.6)	1,226 (6.0)	1,165 (5.7)	962 (4.5)	913 (4.1)	0.548	0.001
	Carbomer	469 (3.5)	606 (3.9)	682 (3.9)	495 (2.4)	623 (3.0)	489 (2.3)	439 (2.0)	0.548	0.012
	Other	249 (1.9)	224 (1.4)	258 (1.5)	221 (1.1)	212 (1.0)	81 (0.4)	110 (0.5)	-	-
NSAID	Pranoprofen	1,313 (9.9)	1778 (11.5)	2,441 (13.8)	3,222 (15.9)	3,336 (16.3)	2,627 (12.3)	3,171 (14.4)	0.072	0.148
	Diclofenac	489 (3.7)	604 (3.9)	642 (3.6)	790 (3.9)	1,017 (5.0)	1,623 (7.6)	2023 (9.2)	0.003	0.007
Corticosteroid	Flurometholone	264 (2.0)	348 (2.2)	438 (2.5)	544 (2.7)	641 (3.1)	807 (3.8)	870 (3.9)	0.003	<0.001
	Prednisolone	39 (0.3)	66 (0.4)	60 (0.3)	96 (0.5)	130 (0.6)	148 (0.7)	148 (0.7)	0.010	0.003
	Other	19 (0.1)	4 (0.0)	2 (0.0)	0 (0.0)	13 (0.1)	44 (0.2)	39 (0.2)	-	-
Vitamin-A preparation	Vitamin-A palmitate	219 (1.6)	321 (2.1)	333 (1.9)	330 (1.6)	327 (1.6)	358 (1.7)	459 (2.1)	0.036	0.879
Immunosuppressant	Cyclosporine	2 (0.0)	2 (0.0)	0 (0.0)	0 (0.0)	0 (0.0)	0 (0.0)	0 (0.0)	-	-
	Tacrolimus	0 (0.0)	0 (0.0)	0 (0.0)	1 (0.0)	0 (0.0)	0 (0.0)	2 (0.0)	-	-

Data are expressed as prescription number (percent of total prescriptions). Artificial tears are expressed by major ingredients. PVA: polyvinyl alcohol. HPMC: hydroxy propyl methyl cellulose. CMC: carboxyl methyl cellulose. PEG: polyethylene glycol. P_1_, *p*-value for trend in number of prescriptions, assessed by Mann–Kendall trend test; P_2_, *p*-value for trend in proportion of prescriptions, assessed by log-linear analysis.

**TABLE 3:** Cost of topical drugs for the treatment of dry eye disease.

Drug class	Drug	2013	2014	2015	2016	2017	2018	2019	P_1_	P_2_
Lubricant	Sodium hyaluronate	529,991 (35.6)	550,109 (29.9)	676,814 (30.9)	890,512 (34.5)	959,859 (36.9)	1,029,898 (40.8)	1,075,786 (41.1)	0.003	0.043
	PVA	144,385 (9.7)	239,248 (13.0)	338,354 (15.5)	409,161 (15.9)	335,415 (12.9)	309,423 (12.3)	316,742 (12.1)	0.548	0.716
	HPMC	94,935 (6.4)	116,954 (6.4)	121,819 (5.6)	115,754 (4.5)	110,219 (4.2)	47,464 (1.9)	40,848 (1.6)	0.133	0.003
	CMC	72,236 (4.8)	169,387 (9.2)	194,181 (8.9)	141,559 (5.5)	131,788 (5.1)	84,128 (3.3)	61,511 (2.3)	0.230	0.053
	PEG	70,620 (4.7)	77,082 (4.2)	84,856 (3.9)	85,507 (3.3)	80,774 (3.1)	58,418 (2.3)	56,326 (2.2)	0.548	<0.001
	Carbomer	25,451 (1.7)	32,403 (1.8)	36,812 (1.7)	23,540 (0.9)	29,155 (1.1)	20,366 (0.8)	18,017 (0.7)	0.133	0.003
	Other	8,512 (0.6)	8,581 (0.5)	10,827 (0.5)	9,917 (0.4)	8,923 (0.3)	1,454 (0.1)	1,662 (0.1)	-	-
NSAID	Pranoprofen	89,325 (6.0)	120,933 (6.6)	164,023 (7.5)	217,887 (8.4)	221,848 (8.5)	143,050 (5.7)	165,911 (6.3)	0.133	0.997
	Diclofenac	13,472 (0.9)	18,142 (1.0)	18,677 (0.9)	34,029 (1.3)	46,014 (1.8)	79,506 (3.1)	102,670 (3.9)	0.003	0.002
Corticosteroid	Flurometholone	5,539 (0.4)	7,718 (0.4)	10,120 (0.5)	12,100 (0.5)	14,729 (0.6)	16,335 (0.6)	17,647 (0.7)	0.003	<0.001
	Prednisolone	1,350 (0.1)	2,324 (0.1)	2,196 (0.1)	3,293 (0.1)	4,166 (0.2)	4,768 (0.2)	5,050 (0.2)	0.007	0.003
	Other	1862 (0.1)	372 (0.0)	120 (0.0)	0 (0.0)	1,020 (0.0)	3,272 (0.1)	17,874 (0.7)	-	-
Vitamin-A preparation	Vitamin-A palmitate	9,494 (0.6)	15,119 (0.8)	14,098 (0.6)	17,586 (0.7)	19,749 (0.8)	17,287 (0.7)	21,970 (0.8)	0.036	0.341
Immunosuppressant	Cyclosporine	300 (0.0)	150 (0.0)	0 (0.0)	0 (0.0)	0 (0.0)	0 (0.0)	0 (0.0)	-	-
	Tacrolimus	0 (0.0)	0 (0.0)	0 (0.0)	779 (0.0)	0 (0.0)	0 (0.0)	3,117 (0.1)	-	-

Data are expressed as cost in Chinese Yuan (percent of total cost). Artificial tears are expressed by major ingredients. PVA: polyvinyl alcohol. HPMC: hydroxy propyl methyl cellulose. CMC: carboxyl methyl cellulose. PEG: polyethylene glycol. P_1_, *p*-value for trend in number of prescriptions, assessed by Mann–Kendall trend test; P_2_, *p*-value for trend in proportion of prescriptions, assessed by log-linear analysis.

NSAIDs are the second largest class of drugs for DED treatment. Pranoprofen was the second most frequently used drug throughout the study, and its use increased both in terms of percentage of prescriptions (*p* < 0.05). Corticosteroid and vitamin A preparation were used by only a small fraction of patients. Flurometholone was the most frequently used corticosteroid, and its use increased progressively in prescriptions and cost (both *p* < 0.05). However, it was used by <5% of patients at the end of the study. There was no significant trend in terms of the percentage of patients using vitamin A palmitate eye gel, the only vitamin A preparation (*p* > 0.05). Cyclosporine and tacrolimus were seldom used.

### Trends in the Use of Drugs for the Treatment of Co-incident Diseases

Yearly prescriptions and cost of drugs for treating co-incident diseases are shown in Table 4 and Figure 2A. The most frequently used drugs for co-incident diseases were antimicrobial agents and growth factor preparations. The former showed an increasing trend in visits (*p* < 0.05), while the latter did not (*p* > 0.05). Notably, the use of anti-allergy and glaucoma drugs increased over time (both *p* < 0.05).

**TABLE 4:** Prescription for specific drug classes used to treat co-incident disease.

Drug class	ATC code	2013	2014	2015	2016	2017	2018	2019	*p*
Anti-microbial agent	S01 A/C	3,877	4,737	4,720	5,394	5,561	6,304	6,357	0.007
Wound healing agent	-	2,141	2,387	2,104	2,330	2028	2,557	2,611	0.368
Antiallergy	S01G	527	520	571	700	772	847	873	0.007
Glaucoma drug	S01E	662	771	921	1,215	1,393	1,615	1,561	0.007
Cataract drug	-	878	883	1,008	1,133	1,168	807	650	>0.999
Complementary medicine	-	126	228	242	356	369	441	410	0.007
Other	-	94	77	99	58	121	170	314	-

*p*-value for trend in number of prescriptions were assessed by Mann–Kendall trend test. Main drugs of wound healing agents are basic fibroblast growth factor preparations and epidermal growth factor preparations. Main drug of cataract drugs is Pirenoxine Sodium.

### Trends in Treatment Patterns

Trends in treatment patterns are shown in Figure 2B. Monotherapy and dual therapy were used in about 80% of prescriptions. However, the fractions of each treatment pattern showed no significant trends during the study period (all *p* > 0.05) and treatment patterns had changed. The most frequently used drug combination in each year of the study was sodium hyaluronate and pranoprofen.

## Discussion

This is the first study evaluating the patterns and trends of DED topical presscriptional treatment in old patients. As data were derived from many hospitals located in six major areas of China, the results are representative of the aged Chinese population. We found that the prescription numbers and cost of DED treatment increased from 2013 to 2019. Ocular lubricants were the major drug used for treatment, and sodium hyaluronate eye drops were the most frequently prescribed drug.

The growing number of yearly prescriptions may reflect an increasing prevalence of DED in the Chinese population. It is reported there is no significant difference in prevalence rate of urban China and rural China (Liu et al., 2014). Although the included hospitals mainly locate in major cities, sampling bias may be neglected. Age was shown to be a risk factor for DED (Song et al., 2018; Dana et al., 2019b), and our study was focused on patients over the age of 60 years. Patients aged between 61 and 70 years were the major part and kept on increasing. This may be associated with the increased use of electronic devices (Courtin et al., 2016). Other possible reasons for the increase in yearly prescriptions include greater awareness of DED among doctors, improvements in diagnostic technologies, and higher demand for care. There were more prescriptions for female patients than for male patients, and this finding is consistent with the reported sex disparity in DED prevalence (Dana et al., 2019b; Siffel et al., 2020).

Ocular lubricants are designed to support the quality and quantity of tear film and are the first-line treatment for DED in many countries (Dogru et al., 2013; Jones et al., 2017). It was also the main drug used to treat DED in China, in contrast to the United States where the more costly cyclosporine is most frequently used (Clayton, 2018; Seitzman and Lietman, 2018). Moreover, many ocular lubricants are over-the-counter drugs and patients can get these drugs from community pharmacy, and the use rate of ocular lubricant may be higher than the result of our study. Nearly all ocular lubricant formulations can relieve DED symptoms and may improve visual acuity and protect against ocular damage (Moshirfar et al., 2014). However, randomized trials of their efficacy have been limited by a small sample size and poor study design (Pucker et al., 2012). Most ocular lubricant formulations have similar efficacy (Pucker et al., 2012), although CMC-, HPMC-, and hyaluronate-based formulations have been shown to be the most effective in improving patient comfort levels (White et al., 2014). These three formulations accounted for the majority of prescriptions in our analysis, with the hyaluronate-based formulation being the most frequently prescribed (Table 2). Ocular lubricants have good safety profiles and ophthalmologists may take this into consideration when prescribing for old patients. However, the reasons for its widespread use as well as its pharmaco-economic profile require further investigation. There is no evidence for the superior efficacy of PVA-based formulations (Nelson and Farris, 1988; McDonald et al., 2002); however, their prescription increased over the study period and ranked second in terms of cost among all DED drugs. Perhaps this growth could be attributed to more aggressive marketing efforts.

A variety of topical NSAID formulations have been used to treat DED. NSAIDs were the second largest class of drugs for DED treatment in this study, and their use increased progressively in both prescription numbers and cost. The most frequently studied NSAIDS in literatures are pranoprofen, diclofenac acid, ketorolac, and indomethacin (Rolando et al., 2002; Aragona et al., 2005; Chen et al., 2014). Only two of these—pranoprofen and diclofenac—were among the drugs prescribed in our study. Results from clinical trials have shown that some NSAIDs—especially diclofenac—can reduce corneal sensitivity in DED patients; moreover, diclofenac suppressed hyperosmolarity-induced apoptosis of corneal cells (Sawazaki et al., 2014). However, more studies are needed to determine which NSAID is the most effective (Aragona et al., 2005). The increased use of topical NSAIDs warrants attention as sporadic cases of corneal melting have been reported in DED patients (Isawi and Dhaliwal, 2007). Moreover, there is little known about the effects of long-term topical NSAID use, as the treatment duration was no more than 1 month in most studies (Jones et al., 2017). Additionally, the reason for the frequent use of pranoprofen should be investigated as it is not normally recommended in treatment guidelines.

Topical glucocorticoid eye drops can effectively relieve signs and symptoms of DED (Thulasi et al., 2017), but there are adverse effects, such as glaucoma and cataract, associated with long-term corticosteroid use. Thus, topical corticosteroid should be used cautiously, and pulse treatment is a common option. Unsurprisingly, topical corticosteroid accounted for a small fraction of prescriptions in our study. Among corticosteroids, fluorometholone, and loteprednol have a lower risk of increasing intraocular pressure and inducing cataract formation (Mataftsi et al., 2011; Sheppard et al., 2016; Jones et al., 2017). Nearly 80% of the corticosteroid prescriptions in China were for fluorometholone; thus, the current evidence supports the clinical application of corticosteroids.

Topical cyclosporine was the first drug approved for DED treatment and is widely prescribed by ophthalmologists in North America (Clayton, 2018). It was previously reported that cyclosporine accounted for 99% of the total expenditure for DED drugs (Seitzman and Lietman, 2018); however, we found that it is rarely used in China. Asian countries have a different view of DED etiology from that of the United States, recognizing tear instability rather than inflammation as the main cause for DED (Watanabe, 2018). Uncertain efficacy, side effects, and high cost also limit the use of these drugs (de Paiva et al., 2019; White et al., 2019; White et al., 2020).

In addition to drugs for DED treatment, patients may be using other drugs for co-incident disease. Our analysis showed that while drugs for DED represented the largest proportion of the total cost, anti-microbial agents, growth factor preparations, and anti-allergy agents were frequently prescribed. This result is in accordance with epidemiologic findings that microbial infection, surgery/corneal ulcer, and allergic disease are common comorbidities of DED (Shimazaki et al., 2020). The high number of visits for glaucoma drugs among DED patients may indicate the growing prevalence of this comorbidity, which should be noted by clinicians (Dana et al., 2019a).

Monotherapy was implemented in less than half of the patients observed in our study, which suggests that the standard therapeutic approach did not yield satisfactory outcomes in most cases. The level of patient-reported satisfaction with over-the-counter formulations including hyaluronate is about 64% (Gomes and Santo, 2019). However, the efficacy of hyaluronate plus pranoprofen—the most frequently used drug combination for DED treatment—is not supported by clinical evidence, despite the proven efficacy of each drug as monotherapy (McDonald et al., 2002; Chen et al., 2014). This raises a concern for the overuse of these drugs. Combinations of three or more drugs are restricted by the need for frequent administration.

This study had some limitations. Firstly, the outcomes of DED treatment with eye drops were not documented in the database used in our study. And they require more detailed investigation. Secondly, the cohort was not stratified by DED phenotype or severity. Thirdly, we were unable to determine whether the topical formulations contained preservatives based on the available information. Finally, we did not include oral drugs and non-prescription drugs for DED treatment in our analysis.

## Conclusion

We analyzed trends in DED topical prescriptional treatment over a seven -year period in old Chinese patients. The prescription numbers and corresponding cost associated with DED both showed increasing trends over the study period, highlighting the need for better clinical management of old patients with DED. Ocular lubricants were the most frequently used drug for DED treatment, and this tendency may reflect the view among Chinese physicians that tear instability is main cause of DED.

## Data Availability

The original contributions presented in the study are included in the article/Supplementary Material, further inquiries can be directed to the corresponding authors.
